# Supplementary material for: Can Use of Digital Technologies by People with Dementia Improve Self-Management and Social Participation? A Systematic Review of Effect Studies
Source: J Clin Med. 2021 Feb 5;10(4):604. doi: 10.3390/jcm10040604 (PMC7915697; doi:10.3390/jcm10040604)
Supplement: Supplementary file 1 [file jcm-10-00604-s001.zip › jcm-1061476-supplementary.pdf]

Supplementary - Search Strings

PubMed

<u>Dementia</u>

"Dementia"[Mesh] OR "Cognitive Dysfunction"[Mesh] OR dement*[tiab] OR Alzheimer*[tiab] OR mild cognitive impairment*[tiab] OR ((MCI[tiab] AND mild[tiab]) OR (MCI[tiab] AND cognitive[tiab]))

<u>Computer assisted technology (App/tablet)</u>

"Internet"[Mesh] OR "Information Technology"[Mesh] OR "Self-Help Devices"[Mesh] OR "Mobile applications"[Mesh] OR "Therapy, Computer-Assisted"[Mesh] OR "Microcomputers"[Mesh] OR "Telemedicine"[Mesh] OR "Virtual Reality"[Mesh] OR "Augmented Reality"[Mesh] OR mobile application*[tiab] OR Computer-assisted[tiab] OR computer-based[tiab] OR Web-based[tiab] OR E-health[tiab] OR mobile health[tiab] OR mobile phone*[tiab] OR Smartphone*[tiab] OR smart phone*[tiab] OR Mobile device*[tiab] OR Tablet[tiab] OR mobile app[tiab] OR m-health[tiab] OR ehealth[tiab] OR mhealth[tiab] OR iPad*[tiab] OR iPhone*[tiab] OR blended[tiab] OR telemedicine*[tiab] OR telerehabilitation*[tiab] OR telepsychiatry[tiab] OR telepsychology[tiab] OR teleconsultation*[tiab] OR remote consultation*[tiab] OR software-app*[tiab] OR electronic-app*[tiab] OR handheld-device*[tiab] OR hand-held-device*[tiab] OR handheld-computer*[tiab] OR hand-held-computer*[tiab] OR microcomputer*[tiab] OR palmtop*[tiab] OR laptop*[tiab] OR personal digital assist*[tiab] OR PDA[tiab] OR telephone application*[tiab] OR touch-screen*[tiab] OR touchscreen*[tiab] OR assistive technolog*[tiab] OR Robot*[tiab] OR wearable*[tiab] OR virtual realit*[tiab] OR augmented realit*[tiab] OR exergam*[tiab]

<u>Social health</u>

"Social Determinants of Health"[Mesh] OR "Activities of Daily Living"[Mesh] OR "Empowerment"[Mesh] OR "Social Isolation"[Mesh] OR "Loneliness"[Mesh] OR "Personal Autonomy"[Mesh] OR Social Health[tiab] OR Self-efficacy[tiab] OR Self-management[tiab] OR Social participation[tiab] OR Experienced Autonomy[tiab] OR Autonomy[tiab] OR Social activit*[tiab] OR social contact*[tiab] OR Social obligation*[tiab] OR meaningful activit*[tiab] OR activities of daily living[tiab] OR occupational function*[tiab] OR lonel*[tiab] OR psychosocial[tiab] OR wellbeing[tiab] OR well-being[tiab] OR social isolation*[tiab] OR empowerment[tiab] OR self-care skill*[tiab] OR self-determination[tiab]

Date filter:

("2007/01/01"[EDAT]: "3000/01/01"[EDAT]) OR ("2007/01/01"[MHDA]: "3000/01/01"[MHDA]) OR ("2007/01/01"[PDAT]: "3000/01/01"[PDAT])

Cinahl (EBSCO)

<u>Dementia</u>

(MH "Dementia+") OR TI(dement* OR Alzheimer* OR "mild cognitive impairment*" OR ((MCI AND mild) OR (MCI AND cognitive))) OR AB(dement* OR Alzheimer* OR "mild cognitive impairment*" OR ((MCI AND mild) OR (MCI AND cognitive))) OR SU(dement* OR Alzheimer* OR "mild cognitive impairment*" OR ((MCI AND mild) OR (MCI AND cognitive)))

<u># Computer assisted technology (App/tablet)</u>

(MH "Telehealth+") OR (MH "Internet+") OR (MH "Information Technology") OR (MH "Assistive Technology Devices") OR (MH "Mobile Applications") OR (MH "Therapy, Computer Assisted") OR (MH "Microcomputers+") OR (MH "Augmented Reality") OR (MH "Virtual Reality") OR (MH "Remote Consultation") OR TI("mobile application*" OR "Computer-assisted" OR "computer-based" OR "Web-based" OR "E-health" OR "mobile health" OR "mobile phone*" OR Smartphone* OR "smart phone*" OR "Mobile device*" OR Tablet OR "mobile app" OR "m-health" OR ehealth OR mhealth OR iPad*OR iPhone*OR blended OR telemedicine* OR telerehabilitation* OR telepsychiatry OR telepsychology OR teleconsultation* OR "remote consultation" OR "software-app*" OR "electronic-app*" OR "handheld-device*" OR "hand-held-device*" OR "handheld-computer*" OR "hand-held-computer*" OR microcomputer* OR palmtop* OR laptop* OR "personal digital assist*" OR PDA OR "telephone application*" OR "touch-screen*" OR touchscreen* OR "assistive technolog*" OR Robot* OR wearable* OR "virtual realit*" OR "augmented realit*" OR exergam*) OR AB("mobile application*" OR "Computer-assisted" OR "computer-based" OR "Web-based" OR "E-health" OR "mobile health" OR "mobile phone*" OR Smartphone* OR "smart phone*" OR "Mobile device*" OR Tablet OR "mobile app" OR "m-health" OR ehealth OR mhealth OR iPad*OR iPhone*OR blended OR telemedicine* OR telerehabilitation* OR telepsychiatry OR telepsychology OR teleconsultation* OR "remote consultation" OR "software-app*" OR "electronic-app*" OR "handheld-device*" OR "hand-held-device*" OR "handheld-computer*" OR "hand-held-computer*" OR microcomputer* OR palmtop* OR laptop* OR "personal digital assist*" OR PDA OR "telephone application*" OR "touch-screen*" OR touchscreen* OR "assistive technolog*" OR Robot* OR wearable* OR "virtual realit*" OR "augmented realit*" OR exergam*) OR SU("mobile application*" OR "Computer-assisted" OR "computer-based" OR "Web-based" OR "E-health" OR "mobile health" OR "mobile phone*" OR Smartphone* OR "smart phone*" OR "Mobile device*" OR Tablet OR "mobile app" OR "m-health" OR ehealth OR mhealth OR iPad*OR iPhone*OR blended OR telemedicine* OR telerehabilitation* OR telepsychiatry OR telepsychology OR teleconsultation* OR "remote consultation" OR "software-app*" OR "electronic-app*" OR "handheld-device*" OR "hand-held-device*" OR "handheld-computer*" OR "hand-held-computer*" OR microcomputer* OR palmtop* OR laptop* OR "personal digital assist*" OR PDA OR "telephone application*" OR "touch-screen*" OR touchscreen* OR "assistive technolog*" OR Robot* OR wearable* OR "virtual realit*" OR "augmented realit*" OR exergam*)

<u># Social health</u>

(MH "Social Determinants of Health") OR (MH "Autonomy") OR (MH "Patient Autonomy") OR (MH "Social Participation") OR (MH "Activities of Daily Living+") OR (MH "Empowerment") OR (MH "Loneliness") OR (MH "Psychological Well-Being") OR TI("Social Health" OR "Self-efficacy" OR "Self-management" OR "Social participation" OR "Experienced Autonomy" OR Autonomy OR "Social activit*" OR "social contact*" OR "Social obligation*" OR "meaningful activit*" OR "activities of daily living" OR "occupational function*" OR lonel* OR psychosocial OR wellbeing OR "well-being" OR "social isolation*" OR empowerment OR "self-care skill*" OR "self-determination") OR AB("Social Health" OR "Self-efficacy" OR "Self-management" OR "Social participation" OR "Experienced Autonomy" OR Autonomy OR "Social activit*" OR "social

contact*" OR "Social obligation*" OR "meaningful activit*" OR "activities of daily living" OR "occupational function*" OR lonel* OR psychosocial OR wellbeing OR "well-being" OR "social isolation*" OR empowerment OR "self-care skill*" OR "self-determination") OR SU("Social Health" OR "Self-efficacy" OR "Self-management" OR "Social participation" OR "Experienced Autonomy" OR Autonomy OR "Social activit*" OR "social contact*" OR "Social obligation*" OR "meaningful activit*" OR "activities of daily living" OR "occupational function*" OR lonel* OR psychosocial OR wellbeing OR "well-being" OR "social isolation*" OR empowerment OR "self-care skill*" OR "self-determination")

APA PsycInfo (EBSCO)

<u>Dementia</u>

DE "Presenile Dementia" OR DE "Senile Dementia" OR DE "Vascular Dementia" OR DE "Cognitive Impairment" OR DE "Alzheimer's Disease" OR DE "Dementia with Lewy Bodies" OR DE "Dementia" OR TI(dement* OR Alzheimer* OR "mild cognitive impairment*" OR ((MCI AND mild) OR (MCI AND cognitive))) OR AB(dement* OR Alzheimer* OR "mild cognitive impairment*" OR ((MCI AND mild) OR (MCI AND cognitive))) OR KW(dement* OR Alzheimer* OR "mild cognitive impairment*" OR ((MCI AND mild) OR (MCI AND cognitive)))

<u>Computer assisted technology (App/tablet)</u>

DE "Online Therapy" OR DE "Teleconsultation" OR DE "Telepsychiatry" OR DE "Telepsychology" OR DE "Telerehabilitation" OR DE "Computer Assisted Therapy" OR DE "Internet" OR DE "Telemedicine" OR DE "Laptop Computers" OR DE "Microcomputers" OR DE "Computer Applications" OR DE "Virtual Reality" OR DE "Augmented Reality" OR TI("mobile application*" OR "Computer-assisted" OR "computer-based" OR "Web-based" OR "E-health" OR "mobile health" OR "mobile phone*" OR Smartphone* OR "smart phone*" OR "Mobile device*" OR Tablet OR "mobile app" OR "m-health" OR ehealth OR mhealth OR iPad*OR iPhone*OR blended OR telemedicine* OR telerehabilitation* OR telepsychiatry OR telepsychology OR teleconsultation* OR "remote consultation*" OR "software-app*" OR "electronic-app*" OR "handheld-device*" OR "hand-held-device*" OR "handheld-computer*" OR "hand-held-computer*" OR microcomputer* OR palmtop* OR laptop* OR "personal digital assist*" OR PDA OR "telephone application*" OR "touch-screen*" OR touchscreen* OR "assistive technolog*" OR Robot* OR wearable* OR "virtual realit*" OR "augmented realit*" OR exergam*) OR AB("mobile application*" OR "Computer-assisted" OR "computer-based" OR "Web-based" OR "E-health" OR "mobile health" OR "mobile phone*" OR Smartphone* OR "smart phone*" OR "Mobile device*" OR Tablet OR "mobile app" OR "m-health" OR ehealth OR mhealth OR iPad*OR iPhone*OR blended OR telemedicine* OR telerehabilitation* OR telepsychiatry OR telepsychology OR teleconsultation* OR "remote consultation*" OR "software-app*" OR "electronic-app*" OR "handheld-device*" OR "hand-held-device*" OR "handheld-computer*" OR "hand-held-computer*" OR microcomputer* OR palmtop* OR laptop* OR "personal digital assist*" OR PDA OR "telephone application*" OR "touch-screen*" OR touchscreen* OR "assistive technolog*" OR Robot* OR wearable* OR "virtual realit*" OR "augmented realit*" OR exergam*) OR KW("mobile application*" OR "Computer-assisted" OR "computer-based" OR "Web-based" OR "E-health" OR "mobile health" OR "mobile

phone*" OR Smartphone* OR "smart phone*" OR "Mobile device*" OR Tablet OR "mobile app" OR "m-health" OR ehealth OR mhealth OR iPad*OR iPhone*OR blended OR telemedicine* OR telerehabilitation* OR telepsychiatry OR telepsychology OR teleconsultation* OR "remote consultation*" OR "software-app*" OR "electronic-app*" OR "handheld-device*" OR "hand-held-device*" OR "handheld-computer*" OR "hand-held-computer*" OR microcomputer* OR palmtop* OR laptop* OR "personal digital assist*" OR PDA OR "telephone application*" OR "touch-screen*" OR touchscreen* OR "assistive technolog*" OR Robot* OR wearable* OR "virtual realit*" OR "augmented realit*" OR exergam*)

<u># Social health</u>

DE "Empowerment" OR DE "Autonomy" OR DE "Self-Determination" OR DE "Self-Control" OR DE "Activities of Daily Living" OR DE "Self-Care Skills" OR DE "Loneliness" OR TI("Social Health" OR "Self-efficacy" OR "Self-management" OR "Social participation" OR "Experienced Autonomy" OR Autonomy OR "Social activit*" OR "social contact*" OR "Social obligation*" OR "meaningful activit*" OR "activities of daily living" OR "occupational function*" OR lonel* OR psychosocial OR wellbeing OR "well-being" OR "social isolation*" OR empowerment OR "self-care skill*" OR "self-determination") OR AB("Social Health" OR "Self-efficacy" OR "Self-management" OR "Social participation" OR "Experienced Autonomy" OR Autonomy OR "Social activit*" OR "social contact*" OR "Social obligation*" OR "meaningful activit*" OR "activities of daily living" OR "occupational function*" OR lonel* OR psychosocial OR wellbeing OR "well-being" OR "social isolation*" OR empowerment OR "self-care skill*" OR "self-determination") OR KW("Social Health" OR "Self-efficacy" OR "Self-management" OR "Social participation" OR "Experienced Autonomy" OR Autonomy OR "Social activit*" OR "social contact*" OR "Social obligation*" OR "meaningful activit*" OR "activities of daily living" OR "occupational function*" OR lonel* OR psychosocial OR wellbeing OR "well-being" OR "social isolation*" OR empowerment OR "self-care skill*" OR "self-determination")

Web of Science (Clarivate)

<u>Dementia</u>

TS=(dement* OR Alzheimer* OR "mild cognitive impairment*" OR ((MCI AND mild) OR (MCI AND cognitive)))

<u># Computer assisted technology (App/tablet)</u>

TS=("mobile application*" OR "Computer-assisted" OR "computer-based" OR "Web-based" OR "E-health" OR "mobile health" OR "mobile phone*" OR Smartphone* OR "smart phone*" OR "Mobile device*" OR Tablet OR "mobile app" OR "m-health" OR ehealth OR mhealth OR iPad*OR iPhone*OR blended OR telemedicine* OR telerehabilitation* OR telepsychiatry OR telepsychology OR teleconsultation* OR "remote consultation*" OR "software-app*" OR "electronic-app*" OR "handheld-device*" OR "hand-held-device*" OR "handheld-computer*" OR "hand-held-computer*" OR microcomputer* OR palmtop* OR laptop* OR "personal digital assist*" OR PDA OR "telephone application*" OR "touch-screen*" OR touchscreen* OR "assistive technolog*" OR Robot* OR wearable* OR "virtual realit*" OR "augmented realit*" OR exergam*)

<u># Social health</u>

TS=("Social Health" OR "Self-efficacy" OR "Self-management" OR "Social participation" OR "Experienced Autonomy" OR Autonomy OR "Social activit*" OR "social contact*" OR "Social obligation*" OR

“meaningful activit*” OR “activities of daily living” OR “occupational function*” OR lonel* OR psychosocial OR wellbeing OR “well-being” OR “social isolation*” OR empowerment OR “self-care skill*” OR “self-determination”)

Cochrane Central Register of Controlled Trials (CENTRAL) (Wiley)

Dementia

(dement* OR Alzheimer* OR “mild cognitive impairment*” OR (MCI NEAR/3 mild) OR (MCI NEAR/3 cognitive)):ab,ti,kw

Computer assisted technology (App/tablet)

(“mobile application” OR “mobile applications” OR “Computer-assisted” OR “computer-based” OR “Web-based” OR “E-health” OR “mobile health” OR “mobile phone” OR “mobile phones” OR Smartphone* OR “smart phone” OR “smart phones” OR “Mobile device” OR “mobile devices” OR Tablet OR “mobile app” OR “mobile apps” OR “m-health” OR ehealth OR mhealth OR iPad* OR iPhone* OR blended OR telemedicine* OR telerehabilitation* OR telepsychiatry OR telepsychology OR teleconsultation* OR “remote consultation” OR “remote consultations” OR “software-app” OR “software-apps” OR “electronic-app” OR “electronic-apps” OR “handheld-device” OR “handheld-devices” OR “hand-held-device” OR “hand-held-devices” OR “handheld-computer” OR “handheld-computers” OR “hand-held-computer” OR “hand-held-computers” OR “personal digital assistant” OR “personal digital assistants” OR PDA OR “telephone application” OR “telephone applications” OR “touch-screen” OR “touch-screens” OR touchscreen* OR “assistive technology” OR “assistive technologies” OR Robot* OR wearable* OR “virtual reality” OR “virtual realities” OR “augmented reality” OR “augmented realities” OR exergam*):ab,ti,kw

Social health

(“Social Health” OR ”Self-efficacy” OR “Self-management” OR “Social participation” OR “Experienced Autonomy” OR Autonomy OR “Social activity” OR “social activities” OR “social contact” OR “social contacts” OR “Social obligation*” OR “meaningful activity” OR “meaningful activities” OR “activities of daily living” OR “occupational function” OR “occupational functioning” OR “occupational functions” OR lonel* OR psychosocial OR wellbeing OR “well-being” OR “social isolation” OR empowerment OR “self-care skill” OR “self-care skills” OR “self-determination”):ab,ti,kw
